# An Assessment of the Bivalent Vaccine as a Second Booster for COVID-19

**DOI:** 10.3390/vaccines11010079

**Published:** 2022-12-29

**Authors:** Sneh Lata Gupta, Rishi K. Jaiswal

**Affiliations:** 1 Department of Pediatrics, Division of Infectious Disease, School of Medicine, Emory University, Atlanta, GA 30322, USA; 2 Cardinal Bernardin Cancer Center, Department of Cancer Biology, Stritch School of Medicine, Loyola University Chicago, Maywood, IL 60153, USA

In the USA, two monovalent COVID-19 mRNA vaccines are primarily used for vaccination. One is BNT162b2 (Pfizer-BioNTech, Comirnaty) and the other is mRNA-1273 (Moderna, Spikevax); they are administered in two doses and used in the primary series of the vaccine, as well as being the single dose in their first booster [1]. Both mRNA vaccines were initially approved under emergency use authorization (EUA) and recently received full licensing by the Food and Drug Administration (FDA). Both of the mRNA monovalent vaccines are based on the Wuhan strain (ancestral) spike mRNA. In their primary vaccination series, mRNA-1273 was administered in two doses of 100 μg, while BNT162b2 was administered at 30 μg. Both the vaccines’ efficacies were more than 90%, with a durability of 5–6 months after administration [2,3]. The first booster was recommended following the continuing emergence of the SARS-CoV-2 variant after six months of their primary vaccination series. The mRNA-1273 booster was administered as a 50 μg dose (half the initial dose) due to safety concerns, and BNT162b2 was administered as 30 μg. However, the mRNA vaccine effectiveness against the more recent Omicron subvariant BA.4/BA.5 was jeopardized even after getting the first booster [4,5,6]. In the USA, to identify and track novel SARS-CoV-2 variants, the Centers for Disease Control and Prevention (CDC) uses genomic surveillance data, and it was suggested that on 26 November 2022 that the previous Omicron subvariant BA.5 was quickly displaced by dominant BQ.1, BQ1.1., and BF.7 subvariants (https://covid.cdc.gov/covid-data-tracker/#variant-proportions, accessed on 27 December 2022), thus demonstrating the rapid appearance of new mutations in the SARS-CoV-2 virus. As the immune response through neutralizing antibodies induced by vaccination declines over time and new Omicron subvariants emerge with more effective viral fitness and stronger immunity escape from anti-spike antibody responses, the need for variant-customized updated COVID-19 vaccine boosters is increasingly imminent. It is challenging to predict future COVID-19 vaccines and maintain antibody neutralization potential against circulating variants. Considering this, Omicron-containing bivalent vaccine provides an alternative modern approach to minimizing COVID-19-related hospitalizations and deaths. There are many bivalent vaccines under clinical trial, such as mRNA-1273.211, mRNA-1273.617.2, mRNA-1273.213, mRNA-1273.529, and mRNA-1273.214 (clintrials.gov NCT04927065). The FDA authorized Moderna and Pfizer-BioNTech bivalent COVID-19 vaccines for use as a booster dose in the 5-year old or above age group. The Modena and Pfizer-BioNTech bivalent booster containing a 1:1 ratio of Wuhan strain spike mRNA and omicron strain BA.4/BA.5 is recommended two months after their completion of the primary series or first booster vaccination under the EUA category [1]. The Pfizer-BioNTech bivalent vaccine consists of BNT162b2 and BNT162b2 Omi (30 μg) components that include 15 μg ancestral (Wuhan) spike mRNA and 15 μg BA.1 spike mRNA. A 50 μg bivalent vaccine mRNA-1273.214 (consists of 25 μg each of ancestral Wuhan spike and Omicron BA.1 spike mRNAs) was compared with the previously authorized 50 μg mRNA-1273 for their second booster dose administration. The objectives phase 2/3 clinical trial aimed to assess the safety, reactogenicity, and immunogenicity of bivalent vaccine mRNA-1273.214 with monovalent mRNA-1273 28 days after their second booster dose. The bivalent omicron-containing mRNA-1273.214 vaccine had an equivalent safety and reactogenicity profile to the monovalent mRNA-1273 booster vaccine. Furthermore, the mRNA-1273.214 vaccine was better at eliciting a neutralizing antibody response against the Omicron BA.1, BA.4, and BA.5 variants, especially if the person had previous infection exposure status [7]. Another study from a group with a similar study also reported that a bivalent vaccine in their phase 2/3 trial, containing an equal amount of the South African Beta (B.1.351) variant, is safe, immunogenic, and has longer antibody durability [8]. One study measured the neutralization titer using a live virus neutralization test and found that the bivalent vaccine containing BA.5 Omicron spike variant illustrated a broadened and improved neutralization potential against the BA.2.75.2 and BQ.1.1 omicron subvariant [9]. Mice data (pre-clinical study) have also suggested that bivalent SARS-CoV-2 mRNA vaccines enhance the breadth of neutralization titers and protection against the BA.5 Omicron variant. This piece of work evaluated the immunogenicity and protective efficacy of two bivalent vaccines recently authorized for use in Europe and the USA, which contained two mRNAs encoding Wuhan-1 and either BA.1 (mRNA-1273.214) or BA.4/5 (mRNA-1273.222) spike proteins. When administered to K18-hACE2 transgenic mice as a booster seven months after the primary vaccination series with mRNA-1273, the bivalent vaccines induced greater breadth and magnitude of neutralizing antibodies than a monovalent mRNA 1273 booster [10]. Another bivalent vaccine comprising Omicron BA.2 and Delta bivalent LNP-mRNA demonstrated a robust antibody response not only for BA.2 but also for BA.2.12.1, BA.2.75, and BA.5 Omicron subvariants. Similarly, the BNT162b2 bivalent BA.4/5 COVID-19 vaccine also showed higher neutralization titers against BA.4.6, BA.2.75.2, BQ.1.1, and XBB.1 subvariant when tested in participants aged >55 years [11]. These data cumulatively support the decision to use these bivalent vaccines for their second booster [12]. On the other hand, two recent independent studies also suggested that antibody neutralization titers against omicron BA.4/BA.5 were similar after getting a bivalent mRNA vaccine booster to the monovalent vaccine [13,14]. One study indicated that when administering a bivalent vaccine (BNT162b2mRNA Wuhan/Omicron BA.4-5 vaccine), healthcare workers observed more adverse side effects than with the monovalent vaccine (BNT162b2mRNA Wuhan). These reports made everyone mindful of the future decision of taking updated bivalent vaccines and raised red flags concerning safety and reactogenicity [15]. Limitations of all the above-mentioned studies include the trial not being randomized, the sample size being too small, the variant sequences causing COVID-19 not being determined, and follow-up studies being scarce, which ultimately means we do not have information about antibody durability and efficacy post-bivalent vaccine administration. None of the trials were designed to evaluate the effectiveness of the bivalent vaccine. Cases are observed when SARS-CoV-2 infection occurred, even after getting a second booster from both monovalent and bivalent vaccines. No bivalent booster has been authorized for younger kids from 6 months to 4 years of age. This single dose of bivalent vaccine is only approved as a booster and not for the primary vaccination series.

In conclusion, from the above-mentioned results, predicted data from future modeling suggested that getting the boosters out as soon as possible could save millions of lives if the world experienced another winter surge. Hence, the FDA authorized the BA.5 bivalent vaccines ahead of their phase 3 clinical trial results to provide quick access to the public domain based on BA.1 data. The primary purpose of the rapid rollout of bivalent vaccines is to reduce severe COVID-19 disease and related hospitalization, especially in the case of an immunocompromised person. However, we still need to carefully assess its safety and immunogenicity profile. Despite this, the question looking to the future remains around bivalent vaccine immunogenicity and effectiveness against the currently dominating omicron variants, such as BQ.1 and BQ1.1. and BF.7 subvariants.
